# The Protective Role of pVHL in Imiquimod-Induced Psoriasis-like Skin Inflammation

**DOI:** 10.3390/ijms23095226

**Published:** 2022-05-07

**Authors:** Isaí Martínez-Torres, Araceli Tepale-Segura, Octavio Castro-Escamilla, Juan Carlos Cancino-Diaz, Sandra Rodríguez-Martínez, Sonia Mayra Perez-Tapia, Laura C. Bonifaz, Mario Eugenio Cancino-Diaz

**Affiliations:** 1 Departamento de Inmunología, Escuela Nacional de Ciencias Biológicas del Instituto Politécnico Nacional, Plan de Ayala y Prolongación de Carpio, Col. Santo Tomas, Alcaldía Miguel Hidalgo, Ciudad de México C.P. 11340, Mexico; isai_mtz88@hotmail.com (I.M.-T.); tepale.araceli@gmail.com (A.T.-S.); sandrarodm@yahoo.com.mx (S.R.-M.); 2 Unidad de Investigación Médica en Inmunoquímica, Hospital de Especialidades, Centro Médico Nacional Siglo XXI, Instituto Mexicano del Seguro Social, Avenida Cuauhtémoc 330 Col. Doctores, Alcaldía Cuauhtémoc, Ciudad de México C.P. 06720, Mexico; larenmc@msn.com (O.C.-E.); or laura.bonifaza@imss.gob.mx (L.C.B.); 3 Unidad de Investigación en Virología y Cáncer, Hospital Infantil De México Federico Gómez, Dr. Márquez 162. Col. Doctores, Alcaldía Cuauhtémoc, Ciudad de México C.P. 06720, Mexico; 4 Departamento de Microbiologia, Escuela Nacional de Ciencias Biológicas del Instituto Politécnico Nacional, Plan de Ayala y Prolongación de Carpio, Col. Santo Tomas, Alcaldia Miguel Hidalgo, Ciudad de México C.P. 11340, Mexico; jccancinodiaz@hotmail.com; 5 Unidad de Desarrollo e Invstigación en Bioterapéuticos (UDIBI), Escuela Nacional de Ciencias Biológicas, Instituto Politécnico Nacional, Ciudad de México C.P. 11340, Mexico; mayra.perez@udibi.com.mx; 6 Coordinación de Investigación en Salud, Centro Médico Nacional Siglo XXI, Instituto Mexicano del Seguro Social, Avenida Cuauhtémoc 330 Col. Doctores, Alcaldía Cuauhtémoc, Ciudad de México C.P. 06720, Mexico

**Keywords:** psoriasis, von Hippel-Lindau protein (pVHL), hypoxia-inducible factor-1α (HIF-1α), angiogenesis, neutrophils, IL-17

## Abstract

Psoriasis is a chronic inflammatory disease distinguished by an excessive proliferation and abnormal differentiation of keratinocytes. Immune cells, such as T lymphocytes and neutrophils, and inflammatory cytokines, such as Tumor Necrosis Factor-α (TNF-α) and interleukin 17 (IL-17), are essential for maintaining psoriatic lesions. Additionally, a hypoxic milieu present in the skin promotes the expression of transcriptional factor hypoxia-inducible factor-1 alpha (HIF-1α). This protein regulates the expression of angiogenic and glycolytic factors, such as vascular endothelial grown factor and lactate dehydrogenase (LDH), both relevant in chronic inflammation. The von Hippel–Lindau protein (pVHL) is a negative regulator of HIF-1α. Previously, we found that pVHL was almost absent in the lesions of psoriasis patients; therefore, we investigated the impact of rescue pVHL expression in lesional skin. We used the imiquimod-induced psoriasis-like mouse model as an adenoviral vector that allowed us to express pVHL in the skin. Our data show that, in lesional skin, pVHL expression was reduced, whereas HIF-1α was increased. Remarkably, the retrieval of pVHL prevented psoriatic lesions, diminishing erythema, scale, and epidermal and vascular thickness. Furthermore, pVHL expression was capable of reducing HIF-1α, LDH, TNF-α and immune cell infiltration (mainly IL-17^+^ neutrophils). In conclusion, our results demonstrate that pVHL has a protective role to play in the pathophysiology of psoriasis.

## 1. Introduction

Psoriasis is a chronic inflammatory cutaneous disease that generates highly vascularized skin lesions in a plaque form and is characterized by an excessive proliferation and defective differentiation of keratinocytes [1]. The prevalence of psoriasis in the worldwide population is estimated at between 1% and 2%; however, in some countries, this percentage can be higher [2]. The etiology of psoriasis is not completely defined, although it is known that genetic or immune factors, such as mutations in the NF-κB pathway and the infiltration of T lymphocytes and neutrophils capable of producing IL-17 and TNF-α, are crucial players in the activation and aberrant proliferation of keratinocytes [3].

In addition to the inflammatory milieu observed in psoriatic skin, the evidence suggests that hypoxic environments as well as abnormal angiogenesis are crucial for the development of the disease [4]. Hypoxia-inducible factor-1 is a master transcriptional factor composed of HIF-1α and HIF-1β subunits, which are activated in response to low oxygen concentrations to maintain a normoxic microenvironment [5]. Upon hypoxia, cytosolic HIF-1α is stabilized and translocated to the nucleus, where it binds to HIF-1β to promote the expression of pro-angiogenic factors such as vascular endothelial growth factor (VEGF), and glycolytic enzymes such as lactate dehydrogenase (LDH) [5,6].

Evidence suggests that HIF-1α is relevant in psoriasis since in vitro human keratinocytes and HaCaT cells have a high proliferation rate as well as an aberrant differentiation when HIF-1α is increased [7]. In psoriasis patients, increased expression of HIF-1α in relation to mRNA and protein levels was reported in lesional skin compared with healthy tissue [8,9]. Moreover, results from transgenic mice revealed that the overexpression of VEFG generates psoriatic-like lesions [10]. Our group also reported increased expression of this angiogenic factor in psoriatic skin patients [8] that was involved in the abnormal vascularization observed in the tissue. In addition, the regulation of HIF-1α seems to be a determinant in the control of inflammatory conditions, as evidenced by the use of molecules that negatively regulate this transcriptional factor [11].

The von Hippel–Lindau protein (pVHL) is a cytosolic adaptor essential to cell functions, since it is involved in several processes including proliferation, survival [12] and, most importantly, the negative regulation of HIF-1α. In normoxia, HIF-1α is hydroxilated by prolyl hidroxylases that cause its recognition by pVHL, Elongins B and C, Cullin 2 and RBX1 complex. This complex possesses enzymatic activity, which causes the ubiquitylation of HIF-1α and its proteasomal degradation. In contrast, a low oxygen level prevents HIF-1α degradation and, as a consequence, causes it to be transported to the nucleus, promoting the expression of its target molecules [13]. Previously, we analyzed the expression of angiogenic molecules in psoriatic skin, finding that pVHL was present in skin from a healthy subject, but almost absent in psoriasis patients. Conversely, we observed the overexpression of HIF-1α in lesional compared with healthy skin [8]; however, to date, it is unknown if pVHL could play a role in the pathophysiology of psoriasis.

Therefore, in this work, we explored the role of pVHL in psoriatic lesions. To do this, we performed a set of experiments using an imiquimod-induced psoriasis-like mouse model to develop psoriatic lesions and, using an adenoviral vector gene delivery approach, induced pVHL expression. Our results show that in imiquimod-induced psoriatic lesions, pVHL was barely expressed, in contrast to HIF-1α, which was highly expressed. Interestingly, an adenoviral vector infection was able to retrieve pVHL expression in psoriatic skin. Additionally, this recovery considerably reduced skin alterations and the expression of HIF-1α and its downstream angiogenic and glycolytic mediators, as well as inflammatory cytokines. Finally, the presence of pVHL decreased the numbers of IL-17^+^ neutrophils infiltrating the lesions. Thus, our results strongly suggest that pVHL has a protective function in the pathophysiology of psoriasis.

## 2. Results

### 2.1. pVHL Expression Almost Absent in the Skin of Imiquimod-Induced Psoriasis-like Skin Inflammation Model

**Figure 1:**
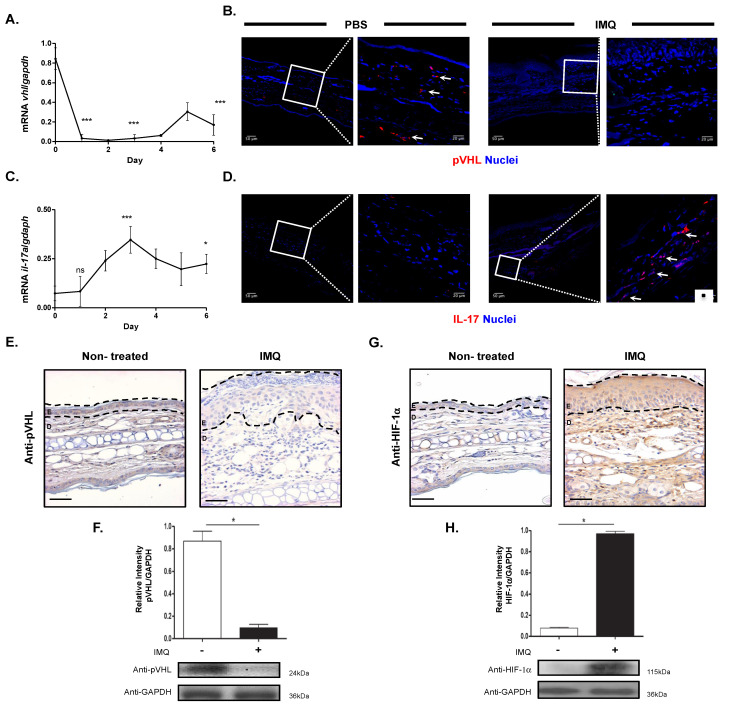
Psoriatic skin from imiquimod-induced psoriasis mice exhibits low presence of pVHL. Balb/c mice were topically treated on the ear with imiquimod cream (5%) over six days. Analyses of mRNA expression of (**A**) vhl and (**C**) il-17a and GAPDH (housekeeping gene) genes in lesional skin using RT-PCR (*n* = 3), one-way ANOVA with post hoc Dunnet’s test. Immunofluorescence images show (**B**) pVHL and (**D**) IL-17 expression in normal and lesional skin. White square indicates the zoomed region. Images are representative of three independent experiments and arrows depict cells expressing pVHL or IL-17. Representative images of immunohistochemistry from lesional skin (E: epidermis, **D**: dermis) performed with (**E**) anti-pVHL or (**G**) anti-HIF-1α. The dashed black lines show epidermal thickness (*n* = 4), Mann–Whitney U two-tailed test. Scale bar = 100 μm. Western blot of (**F**) pVHL and (**H**) HIF-1α expression in lesional skin of mice. Bar graphs show the results from four independent experiments and images are representative of band intensity obtained. * *p* > 0.05, *** *p* > 0.001. IMQ: Imiquimod, pVHL: von Hippel-Lindau protein, HIF-1α: Hypoxia-Inducible Factor-1 alpha, GAPDH: Glyceraldehyde-3-Phosphate Dehydrogenase.

In a previous study, we reported that, in lesional skin from psoriasis patients, the expression of von Hippel–Lindau protein mRNA was low compared with skin from healthy subjects [8]. Here, we worked with an imiquimod-induced psoriasis-like skin inflammation model, widely used to develop psoriatic-like lesions, to evaluate the possible role of pVHL in this pathology. The expression of VHL mRNA in the skin was observed before treatment. However, between the first and third day of imiquimod treatment, we noted a significant reduction in VHL mRNA expression. From day 4 to day 6, we found slight expression of this messenger; however, the expression was much lower compared with normal skin at day 0 (Figure 1A), which is similar at the protein level (Figure 1B). In the same samples, we also evaluated the expression of IL-17, which is an inflammatory cytokine present in psoriatic lesions [3]. Interestingly, when the expression of VHL mRNA was almost absent, the expression of IL-17 mRNA began to increase, reaching maximum expression at day 3, which was maintained until day 6 of treatment (Figure 1C). In addition, immunofluorescence images show similar results, where IL-17 was increased after IMQ treatment at day 6 (Figure 1D).

To confirm these results, we also evaluated the expression of VHL at the protein level in lesional skin of mice using immunohistochemistry and Western blot techniques. We observed that pVHL was present in the dermis and epidermis of normal skin, in contrast to psoriatic lesions (day 6), in which the presence of pVHL was almost absent in both skin layers (Figure 1E). In addition, we observed similar results using Western blot analysis, where we found high presence of pVHL at day 0, but very low presence at day 6 (Figure 1F). These results show that the expression of pVHL is significantly decreased in psoriatic lesions compared with normal skin, resembling the findings in psoriasis patients [8,9]. Considering that pVHL is a negative regulator of HIF-1α [13], we next evaluated the expression of this transcriptional factor at the protein level. Interestingly, we found low presence of HIF-1α in normal skin, while in contrast, at day 6 of the imiquimod treatment we observed high expression in lesional skin (Figure 1G). Furthermore, Western blot analysis revealed low expression of HIF-1α at day 0, which later increased at day 6 of treatment (Figure 1H). Together, these results show that pVHL is expressed in normal skin in contrast to psoriatic skin, where it is almost absent.

### 2.2. Adenoviral Vector Infection Can Induce the Overexpression of the von Hippel–Lindau Protein in Healthy Skin

**Figure 2:**
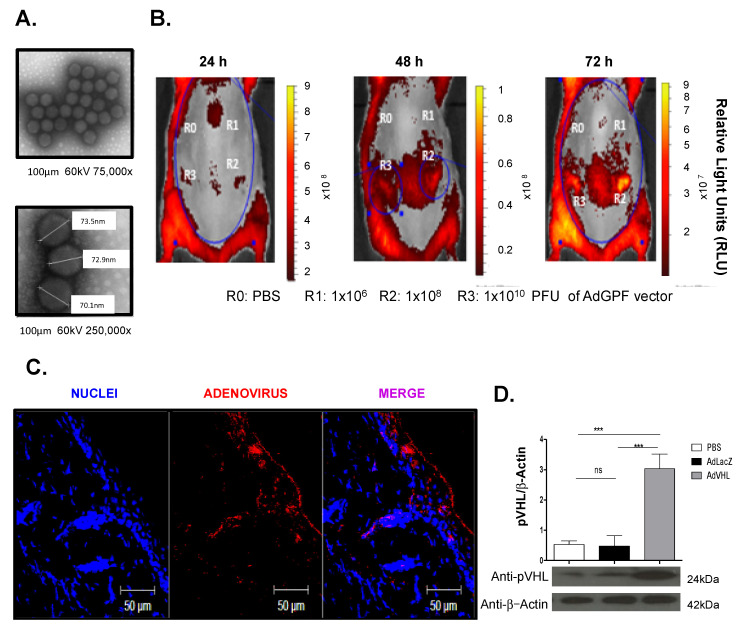
Expression of pVHL and GFP in normal skin of mice treated with adenoviral vectors. (**A**) Representative transmission electron micrographs of adenoviral particles that display a hexagonal shape of approximately 70 nm, 75,000× (upper image) or 250,000× (lower image) magnification. (**B**) Representative images from in vivo imaging of GFP expression. Intradermal injection of R0: PBS, R1: 1 × 10^6^, R2: 1 × 10^8^ or R3: 1 × 10^10^ PFU of AdGFP in four different regions of the backs of mice. GFP expression was evaluated at 24, 48 or 72 h after infection; color bar indicates the intensity of relative light units (RLU). (**C**) Representative confocal micrographs from skin infected with 1 × 10^10^ UFP of AdLacZ. Images show the adenoviral particles (red), nuclei (blue) and merge (magenta). (**D**) Western blot analysis of pVHL expression in skin treated with AdLacZ or AdVHL vectors. Bar graphs shows the results obtained from four independent experiments, band intensity images are representative of one experiment and β-actin was used a control load, two-way ANOVA with post hoc Tukey’s test. *** *p* > 0.001. Scale bar = 50 μm. Ad: Adenoviral vector. pVHL: von Hippel-Lindau protein, GFP: Green Fluorescent Protein, PFU: Plaque-Forming units.

To evaluate whether the pVHL could be overexpressed in normal skin, we used an adenoviral vector system approach. For that purpose, we designed two different constructions: one that expressed the murine pVHL (AdVHL) and another that expressed Green Fluorescent Protein (AdGFP). We observed through electronic microscopy that the recombinant particles obtained had perfect integrity, with a hexagonal shape and adenoviral characteristic size (70–90 nm) (Figure 2A). To test the infectivity of the viral particles, we intradermally inoculated the AdGFP construction at three different doses into the backs of healthy mice and evaluated the GFP expression at 24, 48 and 72 h using in vivo fluorescent imaging. Figure 2B shows low expression of GFP in the backs of the mice at 24 h post-infection, while in contrast, at 48 h, we found high expression of GFP after administration of 1 × 10^8^ Plaque Forming Units (PFU) (R2). This result was more evident with a dose of 1 × 10^10^ (R3) PFU, where the expression of GFP was maintained until 72 h post-infection.

In addition, we observed that an adenoviral presence was detected in a large perimeter surrounding the inoculation site, revealing that infection with AdGFP could spread over the backs of the mice and suggesting the great potential for adenoviral particles to infect mouse skin. With these results, we performed an infection with 1 × 10^10^ PFU of AdVHL to evaluate the expression of pVHL in the skin. Interestingly, Figure 2C shows that AdVHL was capable of infecting both dermal and epidermal cells, and remarkably, the expression of pVHL was significantly higher in the skin of infected mice compared with the empty adenoviral vector infection (AdLacZ) or the PBS group (Figure 2D). Altogether, these results reveal that pVHL expression can be overexpressed in the skin of mice through adenoviral infection.

### 2.3. Expression of pVHL Ameliorates Psoriatic Hallmarks in Imiquimod-Induced Psoriasis-like Model

**Figure 3:**
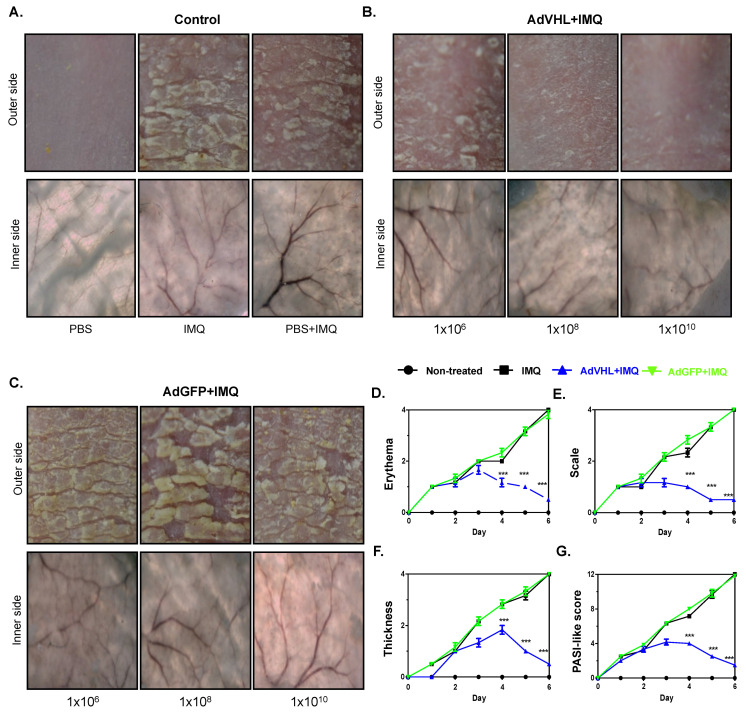
pVHL expression ameliorates the lesions in the imiquimod-induced psoriasis model. Mice were topically treated with 6.25 mg of imiquimod (IMQ) over six days, and skin changes were photographed and scored (*n* = 4). (**A**) Representative images from psoriatic skin (upper panels) and vascularity (lower panels) in mice that only received PBS or IMQ at day 6. Prior to IMQ application (24 h), the mice were injected with 1 × 10^6^, 1 × 10^8^ or 1 × 10^10^ PFU of (**B**) AdVHL or (**C**) AdGFP; in all conditions, psoriatic lesions and vascularity were registered at day 6. Evaluation of (**D**) erythema, (**E**) scale and (**F**) thickness present in skin treated with PBS, IMQ, AdVHL/IMQ or AdGFP/IMQ. Measurements were performed daily during the 6 days of the experiment on each mouse and scored based on skin changes (0: none, 1: slight, 2: moderate, 3: severe, 4: highly severe). (**G**) Psoriasis-like index for the severity of mice psoriasis was calculated by adding erythema, scale and thickness observed in the skin. Data are represented as median and *p* value was calculated using the Kruskal–Wallis test. *** *p* < 0.001. Ad: Adenoviral vector, GFP: Green fluorescent protein, IMQ: Imiquimod, VHL: von Hippel-Lindau.

It has been proposed that pVHL can act as a negative regulator of the transcriptional factor HIF-1α, which was proven to have a role in the pathogenesis of psoriasis [7,11]. Considering that pVHL expression is almost absent in psoriasis lesions in both humans [6] and mice (Figure 1), we rescued pVHL expression and evaluated the effects in psoriasis features in the imiquimod-induced psoriasis-like model. Therefore, we performed a set of experiments applying imiquimod in mice for 6 days to induce psoriatic lesions (control group). In addition, 24 h before the imiquimod application, some mice were intradermally inoculated with AdVHL or AdGFP particles at three different doses to evaluate the development of psoriatic lesions. Figure 3A shows representative photographs of the backs of mice at day 6 of imiquimod treatment. We observed the presence of skin lesions with substantial erythema, scale and thickness. Interestingly, the mice that received AdVHL, even at lower doses (1 × 10^6^ PFU), showed less skin alterations (Figure 3B, upper panel), and remarkably, the mice treated with 1 × 10^8^ and 1 × 10^10^ PFU showed a significant reduction in skin lesions compared with imiquimod treatment without AdVHL. In contrast, the mice treated with AdGFP developed psoriatic lesions in a similar fashion to the imiquimod group (Figure 3C, upper panel). In light of the differences observed in lesional skin due to the rescue of pVHL expression, we then decided to evaluate the morphological changes in the inner side of the skin. The imiquimod-treated group showed an increased presence of dilated blood vessels, suggesting increased angiogenesis (Figure 3A, lower panels). In contrast, the inner skin from the mice treated with imiquimod and 1 × 10^8^ or 1 × 10^10^ PFU of AdVHL showed an important reduction in dilated blood vessels as well as a decreased thickness (Figure 3B, lower panel). This reduction was not observed in the mice treated with AdGFP, where the blood vessels resembled those observed in the imiquimod group (Figure 3C, lower panel). In humans, the severity of psoriasis is calculated based on the Psoriasis Area and Severity Index (PASI) score [1], which measures erythema, scale, and thickness of the affected area of patients. Thus, to determine the severity of the disease in the mouse model, we calculated a PASI-like index in the different groups of mice. Interestingly, we found that the affected area of the mice in the imiquimod group had increased erythema, scale, and thickness during the first two days of treatment, and these features increased drastically after day 3 until day 6 of imiquimod application. As can be observed in Figure 3B, the mice inoculated with AdVHL showed a significant reduction in all severity parameters compared with the imiquimod control group or with the group treated with AdGFP (Figure 3D–F). In addition, the PASI-like index confirmed our results, as the imiquimod and AdGFP mice groups had high scores compared with the mice treated with AdVHL viral particles (Figure 3G). These results show that the expression of pVHL ameliorated the psoriatic hallmarks in the imiquimod-induced psoriasis-like model.

### 2.4. HIF-1α and Its Downstream Mediators in Lesional Skin Are Negatively Regulated by pVHL

**Figure 4:**
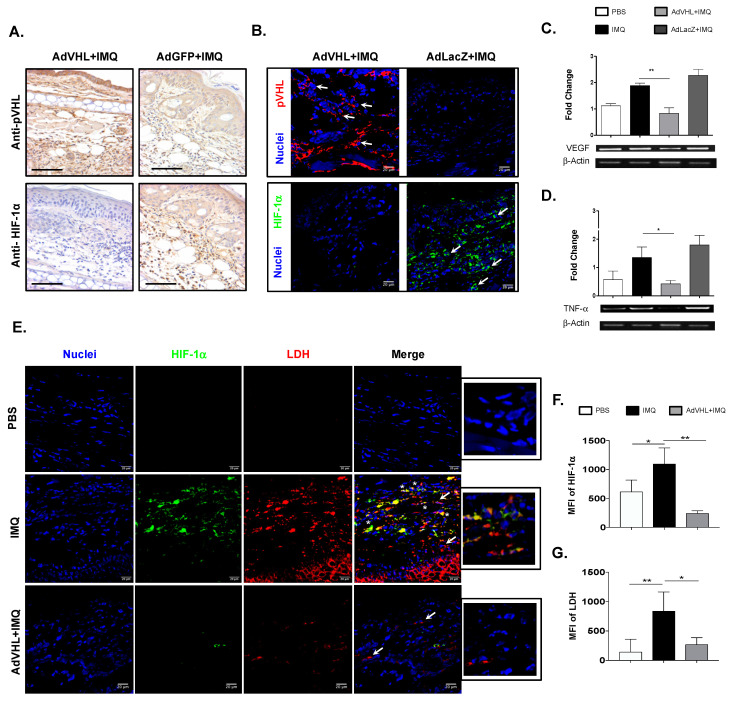
Negative regulation of HIF-1α and its downstream mediators mediated by pVHL expression in psoriatic skin. Immunohistochemistry of mouse ear skin treated with IMQ in the presence of AdVHL or AdGFP. Representative images from lesional skin sections for the detection of: (**A**) pVHL and HIF-1α. Scale bar = 100 μm. (**B**) Immunofluorescence images of pVHL (red), HIF-1α (green) and nuclei (blue) from mice skin treated with AdVHL+IMQ or AdLacZ+IMQ. The images are representative of three independent experiments. RT-PCR analyses from whole lesional skin extract of mice treated with PBS, IMQ, AdVHL+IMQ or AdGFP+IMQ. (**C**) Expression of VEGF and (**D**) TNF-α. Bar graphs show data obtained from six independent experiments (two-way ANOVA with post hoc Tukey’s test), and band images are representative of one experiment. (**E**) Confocal representative images of HIF-1α (green) and LDH (red) expression in normal and lesional skin of mice treated with PBS, IMQ or AdVHL+IMQ. Arrows indicate the cells expressing one molecule of interest, whereas asterisks indicate co-expression of HIF-1α and LDH. Small images depict a digital magnification of the merged pictures. Bar graphs show the Mean Fluorescence Intensity (MFI) of (**F**) HIF-1α and (**G**) LDH expression in the three groups of mice. MFI was evaluated in eight different fields from two independent experiments. One-way ANOVA with post hoc Tukey’s test. * *p* < 0.05, ** *p* < 0.01. Ad: Adenoviral vector, IMQ: Imiquimod, pVHL: von Hippel-Lindau protein, HIF-1α: Hypoxia-Inducible Factor-1 alpha, TNF-α: Tumor Necrosis Factor alpha, VEGF: Vascular Endothelial Growth Factor.

To elucidate the mechanism underlying the inhibition of psoriatic features in the mouse model caused by the retrieval of pVHL, we determined the expression of pVHL in lesional skin from imiquimod-induced psoriasis-like mice, as well as the expression of its target molecule HIF-1α. Figure 4A shows that the epidermal and dermal cells expressed pVHL after imiquimod-AdVHL treatment, compared with the lesional skin cells from mice treated with AdGFP. Importantly, we observed very low expression of HIF-1α in the skin of mice treated with AdVHL, whereas in the lesional skin administered with imiquimod-AdGFP, we observed a high presence of HIF-1α^+^ cells (Figure 4A, lower panel). To confirm these results, we also determined the expression of pVHL and HIF-1α in the presence or absence of AdVHL using immunofluorescence and confocal microscopy. We found similar results, as the lesional skin treated with AdVHL showed high expression of pVHL and limited presence of HIF-1α, while in lesional skin treated with the empty vector (AdLacZ) we found the opposite expression (Figure 4B). These data strongly suggest that pVHL acts as a negative regulator of HIF-1α in this psoriasis model context.

Considering our hypothesis and that this transcriptional factor acts in hypoxic conditions and participates in angiogenesis and inflammation [14], we therefore evaluated the mRNA expression of the pro-angiogenic molecule VEGF, which is positively regulated by HIF-1α as well as TNF-α, a pro-inflammatory cytokine increased in psoriatic skin [6]. The RT-PCR results show that the expression of the VEGF gene in lesional skin increased with imiquimod treatment compared with normal skin. Interestingly, when pVHL was present, we observed a significant decrease in VEFG gene expression compared with imiquimod and AdGFP treatment (Figure 4C). In a similar fashion, we found high TNF-α gene expression in the group that received imiquimod; however, the presence of pVHL substantially reduced the expression of the TNF-α gene (Figure 4D). In addition to angiogenesis and cytokines, glycolysis is another process that has been associated with increased inflammatory response [15]. In order to strengthen our data concerning the negative role of pVHL in the HIF-1α pathway, we evaluated the expression of lactate dehydrogenase, which is a glycolytic enzyme positively regulated by HIF-1α [5], in presence of pVHL. As observed in Figure 4E, the expression of HIF-1α and LDH was almost absent in normal skin, whereas IMQ treatment highly induced the expression of both molecules; in addition, there were several skin cells expressing HIF-1α and LDH simultaneously. Remarkably, confocal microscope images reveal that lesional skin from mice treated with AdVHL had significantly diminished expression of HIF-1α and LDH compared with IMQ-treated skin (Figure 4F,G). Altogether, these results strongly suggest that pVHL contributes to the absence of lesions through the negative regulation of HIF-1α expression and its downstream angiogenic and glycolytic molecules as well as pro-inflammatory cytokines.

### 2.5. Expression of pVHL in Psoriatic Skin Prevents the High Infiltration of IL-17^+^ Neutrophils

**Figure 5:**
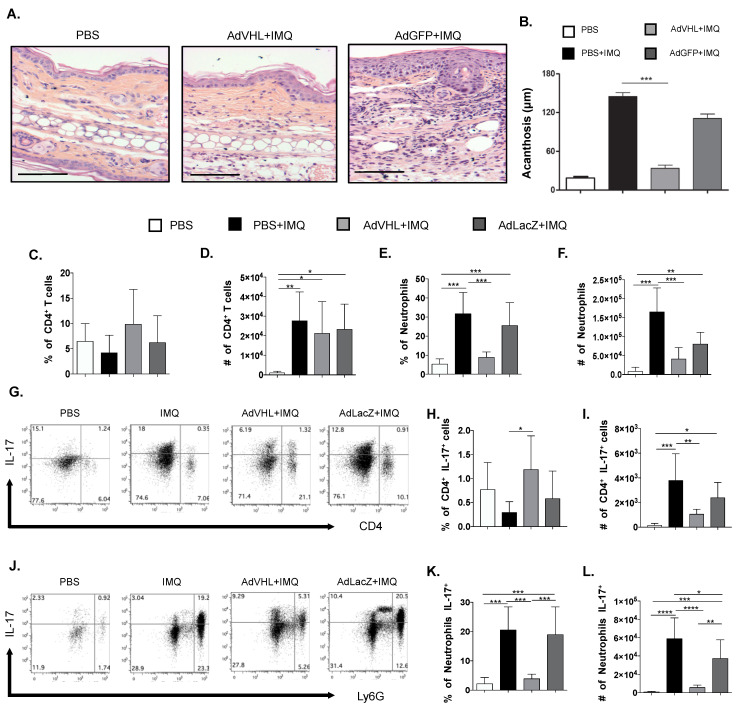
pVHL expression prevents the infiltration of neutrophil IL-17 producers in psoriatic skin. Psoriatic-like lesions were generated by imiquimod (IMQ) application in the ears of mice and received 1 × 10^10^ PFU of AdVHL vector, as described in previous figures. (**A**) Hematoxylin–eosin staining of skin sections from mice treated with PBS, IMQ/AdVHL or AdGFP vectors. Scale bar = 100 μm. (**B**) Bar graphs of measurements of acanthosis observed in ear skin from mice treated as mentioned above. (**C**) Bar graph of percentages and (**D**) total numbers of CD4^+^ T cells. (**E**) Bar graph of percentages and (**F**) total numbers of skin neutrophils. (**G**) Representative dot plots of IL-17 in CD4^+^ T cells (CD4^+^ IL-17^+^) for each treatment. (**H**) Bar graph of percentages and (**I**) total numbers of IL-17 in CD4^+^ T cells (CD4^+^ IL-17^+^) in skin. (**J**) Representative dot plots of IL-17 in neutrophils (Ly6G^+^ IL-17^+^) for each treatment. (**K**) Bar graph of percentages and (**L**) total numbers of IL-17 in neutrophils (Ly6G^+^ IL-17^+^) in the skin. Mean ± SD, *n* = 6–8, data pooled from three independent experiments. One-way ANOVA with Tukey’s multiple comparisons test. * *p* < 0.05, ** *p* < 0.01, *** *p* < 0.001, **** *p* < 0.0001. Ad: Adenoviral vector, GFP: Green fluorescent protein, IMQ: Imiquimod, IL-17: Interleukin 17, VHL: von Hippel-Lindau.

In psoriasis, inflammation and angiogenesis are related to epidermal thickness and cell infiltration in lesional skin [3]. To examine the impact of pVHL rescue on the cell infiltration and acanthosis of psoriatic lesions, we analyzed histological samples from mice treated with imiquimod in the absence of pVHL. In normal skin, hematoxilin–eosin stain revealed few cells infiltrating the dermis and a normal epidermis size. Remarkably, this was similar in the mice treated with AdVHL, indicating less skin inflammation and resembling the lack of psoriatic lesions observed in Figure 3 (Figure 5A,B), while the group treated with AdGFP showed great immune cell infiltration as well as a significant increase in epidermal thickness (Figure 5A,B). These findings indicate that pVHL may diminish cell infiltration and epidermal thickness.

Previous evidence revealed that neutrophils and T cells are important participants in psoriasis lesion microenvironment [3]. In addition, we found that an imiquimod-induced psoriasis-like skin inflammatory model is characterized by increased expression of IL-17 between day 3 and day 6 of imiquimod treatment (Figure 1C,D). Therefore, we explored whether pVHL recovery could influence neutrophil and T cell infiltration as well as IL-17 production in an imiquimod-induced psoriasis context. To achieve this goal, using flow cytometry, we analyzed skin cell suspensions obtained from the lesions of mice. Supplementary Figure S1 shows the identification algorithm, which excludes debris, aggregates and dead cells. We selected CD45^+^ cells to identify leucocytes, and used CD4 and Ly6G as T helper and neutrophil cell lineage markers, respectively.

The results showed no differences in the percentage of CD4^+^ cells in the different groups of mice; however, there was an increased number of T cells in all groups treated with imiquimod (Figure 5C,D). We also found that the percentage and number of neutrophils were increased in the imiquimod group, which shows that neutrophils highly infiltrated the lesional skin but, interestingly, the administration of AdVHL significantly reduced the percentage and number of neutrophils compared with the imiquimod and AdLacZ groups (Figure 5E,F). When we analyzed the production of IL-17 by CD4 T cells, we found a very low percentage of these cells in all groups treated with imiquimod, which was slightly increased in the presence of pVHL. Regarding absolute numbers, CD4 IL-17^+^ cells were increased in the imiquimod and AdLacZ groups compared with the AdVHL group (Figure 5G–I). In contrast, we observed a high frequency and high numbers of neutrophils in the imiquimod group compared with the PBS group. Remarkably, there was a significant reduction in IL-17^+^ neutrophils, both in percentage and number, in the mice treated with AdVHL compared with the imiquimod and AdLacZ groups (Figure 5J–L). These results show that, in psoriatic skin, the expression of pVHL prevents neutrophil migration and its IL-17 production, which may imply a protective role for the von Hippel–Lindau protein in psoriasis.

## 3. Discussion

The von Hippel–Lindau protein is a tumor suppressor gene involved in the negative regulation of HIF-1α in normoxic conditions, whereas mutations on the VHL gene or hypoxic conditions prevent HIF-1α degradation, which promotes the expression of angiogenic and inflammatory mediators [16]. In psoriasis, whether the pVHL/HIF-1α regulatory pathway could play a role in the physiopathology has not been explored. In previous work, we found expression of the VHL gene in healthy skin, in contrast to the lesional skin of psoriasis patients, where its expression is almost absent [8]. Here, we revealed that the reconstitution of pVHL, through an adenoviral vector, could inhibit psoriatic lesion development and reduce angiogenic, inflammatory and glycolytic mediators as well as immune cell infiltration, suggesting a protective function for pVHL in this cutaneous inflammatory disease.

The imiquimod-induced psoriasis skin inflammatory model is widely used to induce psoriatic-like lesions that resemble human disease [17]. Here, we found that pVHL is present in normal skin, with its expression decreasing after the first day of imiquimod treatment and remaining low until the end of the experiment. This result is similar to our previous findings in psoriasis patients [8]. In addition, we discovered increased expression of HIF-1α in lesional skin from psoriatic mice, similar to that in human evidence, which demonstrated that HIF-1α is upregulated in psoriatic lesions compared with normal skin [9,18]. We propose that the deficiency of pVHL in psoriatic skin may be a detrimental factor in this disease. This proposition is also supported by the fact that pVHL was significantly reduced from the first day of imiquimod (IMQ) treatment, which coincided with an increase in IL-17 expression and a high presence of HIF-1α.

In this work, we used genetic engineering to promote pVHL expression using an adenoviral approach. Previous evidence showed that adenoviral gene delivery could be a successful method to overexpress inflammatory and angiogenic molecules in skin cells under in vitro and in vivo conditions [19,20]. We found that infection with AdVHL is effective to induce an overexpression of pVHL in healthy skin and can remarkably overcome its absence in psoriatic skin, where retrieval prevented the appearance of lesions and reduced the severity of the disease features. The aforementioned work also demonstrated that E1-deficient human adenovirus type 5 is capable of infecting human keratinocytes, as well as HaCaT cells [20]. Hence, it is plausible to consider that our AdVHL vector infected keratinocytes in the lesional skin of mice, similar to those found in healthy skin, showing a potential protective role for pVHL in psoriatic skin.

It was previously shown that psoriasis features could be induced using an adenoviral vector encoding IL-23 gene [21]. However, to our knowledge, the potential benefit of adenoviral gene delivery in preventing the appearance of psoriasis lesions has not been explored. Our results strongly suggest that reduced expression of pVHL in the skin is determinant of the development of psoriatic lesions. This proposal is supported by the significant reductions in erythema, scale and epidermal thickness, as well as a minor PASI-like index in mice treated with AdVHL, indicating a reduction in the inflammatory process. Additionally, the considerable reduction in blood vessel dilation confirms the protective role of pVHL in psoriasis. Although the imiquimod-induced psoriasis-like model is a useful tool to study psoriatic events, it is a transitory model, as after seven days of treatment, the mice can resolve the lesions naturally. We considered further experiments to establish whether pVHL could be sufficient to improve and maintain chronic psoriatic lesions.

To date, it is not completely understood how pVHL expression is regulated, although some evidence suggests that it is by epigenetic and RNA silencing mechanisms. In human psoriatic skin, an overexpression of the histone deacetylase 1 (HDAC1) was found [22], which is a negative modulator of pVHL expression [23]. We previously demonstrated that keratinocytes and HaCaT cells transfected with a plasmid that expresses HDAC1 showed a high translocation of HIF-1α to the nucleus with a high production of VEGF [24]. In addition, Frienland et al. showed that topical application of an inhibitor of HDAC1 and HDAC3 (MS-275) reduced the imiquimod-induced hyperproliferation of the keratinocytes and IL-23 expression [25]. This evidence suggests that the low expression of pVHL observed in this study could be related to an increased expression of HDAC1, which also results in the elevation in HIF-1α, VEGF and TNF-α expression observed in psoriatic skin lesions.

Another proposed mechanism to explain the reduced expression of pVHL observed in this study is through non-coding RNAs, particularly microRNAs (miRNAs). It has been reported that a high expression of miRNA21 in glioblastoma cells and papillary thyroid carcinoma tissue can negatively regulate pVHL expression [26,27]. Previous work on humans showed that miRNA21 expression was increased in the epidermis of psoriasis patients. On the other hand, evidence from psoriasis mouse models reveals that inhibition of miRNA21 ameliorates disease pathology [28], and that the miR21-3p strand can regulate the proliferation of keratinocytes and inflammatory response in psoriasis [29]. In addition, it was reported that molecules present in psoriatic skin, such as IL-6, TGF-β and Epidermal Growth Factor, could promote the expression of miRNA21 [30]. Although the mechanism that explains the low expression of pVHL in psoriatic lesions needs to be explored, this is the first study that strongly suggests an important role for pVHL in the pathophysiology of psoriasis.

Moreover, regarding the negative regulation of molecular mediators observed in lesional skin, we also found that the retrieval of pVHL expression decreases the inflammatory cell infiltration in psoriatic skin, particularly in T helper cells and neutrophils. One of the most relevant T helper subsets in psoriasis is the Th17 cells due to their ability to produce IL-17, a pivotal inflammatory cytokine for psoriasis development [31,32]. Our data shows that imiquimod administration slightly increases the numbers of CD4^+^ IL-17^+^ T cells; in contrast, treatment with AdVHL reduces the presence of this population. A seminal work demonstrated that the expression of HIF-1α and STAT3 is necessary for promoting the expression of the master transcriptional factor RORγt, which is essential to establishing a Th17 profile and IL-17 production [33]. Hence, a reduction in HIF-1α expression, as we observed via pVHL retrieval, could inhibit Th17 polarization and ameliorate psoriatic lesions. Beside transcription factors, metabolic processes are determining factors in T cell functionality; for example, glycolysis is associated with a T cell effector state [34]. In the inflammatory milieu, HIF-1α is overexpressed and promotes the expression of glycolytic enzymes such as LDH, which metabolizes pyruvate to generate lactate [35]. It was reported that high lactate concentrations promote the polarization of T CD4+ cells towards Th17 profile, characterized by increased expression of RORγt and high production of IL-17 [36]. Our results show that pVHL retrieval in the lesional skin diminishes the expression of LDH, which could impact Th17 polarization. Therefore, the lower production of IL-17 by CD4 T cells observed in lesional skin resulting from the upregulation of pVHL could be a consequence of the downregulation of HIF-1α and LDH.

In addition, evidence suggests that an absence of pVHL not only affects the function of CD4 T cells, but also other T cell populations that play a relevant role in psoriasis. For instance, it was reported that Treg lymphocytes lacking pVHL expression are capable of producing IFN-γ driven by HIF-1α to develop a spontaneous inflammatory condition [37]. In addition, the absence of pVHL in CD8 T cells leads to an increase in their effector functions as IFN-γ producers and granzymes [38]. Thus, we propose that a lack of pVHL in psoriasis lesions could contribute to the inflammatory milieu through high expression of IL-17 and IFN-γ.

Another important population implicated in psoriasis pathogenesis is neutrophils, which play a role in skin inflammation through Neutrophil Extracellular Traps and cytokine production [39]. Here, we found high neutrophil infiltration in the lesional skin of imiquimod-treated mice. Remarkably, the retrieval of pVHL via treatment with AdVHL reduces neutrophil infiltration in lesions. A previous report by Ward et al. demonstrated that macrophages and neutrophil depletion promoted an improvement in lesional skin in a KC-Tie2 murine model of psoriasis [40]. Furthermore, mice treated with an anti-Ly6G antibody to eliminate neutrophils showed a reduction in psoriatic lesions in an imiquimod-induced psoriasis context [41]. In human psoriasis, Munro’s microabscess in the epidermis comprises neutrophil aggregates, which likely contribute to the inflammatory process [42]. In patients with pustular psoriasis, it has been observed that a depletion in granulocytes and monocytes from blood through plasmapheresis results in a significant improvement in clinical score [43]. Thus, we suggest that pVHL expression in lesional skin can ameliorate psoriasis severity via a reduction in neutrophil infiltration, which could be explained by the reduction in angiogenesis observed in affected skin.

Interleukin 17 is a key factor in psoriasis, mainly produced by T cells and innate lymphocytes as well as neutrophils [31,44]. In this work, we reported that pVHL retrieval in lesional skin after imiquimod application significantly reduces both the production of IL-17 by neutrophils and the severity of disease. Other authors demonstrated that neutrophils are capable of expressing IL-17 in a tape-stripping model [45], or in response to bacterial and fungi components via IL-6 and IL-23 [46,47]. To date, the mechanism that regulates IL-17 production in neutrophils is not completely understood. Nevertheless, as we observed diminished expression of HIF-1α and IL-17 production in the presence of pVHL, we hypothesize that pVHL can exert a negative regulation on IL-17 production through inhibition of the HIF-1α pathway, as previously reported for CD4 T cells [33].

One of the most important strategies for the treatment of psoriasis is focused on blocking IL-17 through the use of monoclonal antibodies that neutralize its function. This biological therapy shows excellent results; however, new therapeutic approaches are under development to improve the quality of life of patients [48]. For instance, inhibitors of the molecules involved in IL-17 signaling or transcriptional factors such as RORγt are undergoing clinical trials and presenting promising results [49]. The adenoviral gene delivery system was used for cancer treatment and, recently, as a COVID-19 vaccine platform, showing excellent results in inducing the expression of proteins in human cells [50,51]. To our knowledge, it has yet to be proposed that an adenoviral vector be applied for psoriasis treatment. Our data strongly suggest that an adenoviral delivery system is a feasible way to induce the expression of pVHL in normal and lesional skin; hence, this brings the possibility of exploring the potential benefit of this approach in psoriasis treatment.

## 4. Materials and Methods

### 4.1. Mice

The Experimental Facility Core of Escuela Superior de Medicina of IPN provided Balb/c mice. All experiments were performed on mice aged between 6 and 8 weeks, in accordance with protocol ZOO-001-2021, and were approved by the Ethical Committee of Escuela Nacional de Ciencias Biologicas. The housing of the mice was according to the Mexican Official Guide (NOM-062-ZOO-1999) for the care and use of laboratory animals. All mice were free of parasites, and fed with LabDiet 5010 Autoclavable Rodent diet (LabDiet, St. Louis, MO, USA) and water ad libitum.

### 4.2. Design and Production of Adenoviral AdVHL, AdGFP and AdLacZ Vectors

Murine VHL gene (Gen Bank NM_009507.3) was obtained by RT-PCR using 5′-CCAATAATGCCCCGGAAGG-3′ (sense) and 5′-TCAAGGCTCCTCTTCCAGGTG-3′ (antisense) primers. cDNA were obtained from cellular RNA extracts of whole skin of healthy mice and were inserted into a Kosak (G/A)NNATGG sequence following insertion in the cloning vector. The *GFP* gene was amplified by PCR from commercial pEGFP plasmid (Invitrogen, Waltham, MA, USA). Both amplified products were cloned into the entry vector pCR^®^8/GW/TOPO^®^ using TA cloning^®^ (Invitrogen, Waltham, MA, USA), and were subsequently subcloned via recombination into the plasmid pAd/CMV/V5-DEST gateway (Invitrogen, Waltham, MA, USA). The recombinants were digested with the Pacl restriction enzyme (ThermoFisher, Waltham, MA, USA) to perform the transfection of the HEK-293A cell line (ATCC, Manassas, VI, USA). After 72 h of culture, the AdVHL and AdGFP vectors were harvested from the supernatant of transfected cells and kept in refrigeration for later amplification and purification. The AdLacZ was a control adenovirus provided by the manufacturer, and was included in cloning kit (Invitrogen, Waltham, MA, USA).

### 4.3. Amplification and Purification of Adenoviral Vectors

The AdVHL, AdGFP and AdLacZ adenoviral vectors were expanded by infection cycles for the 293A cell line (ATCC, Manassas, VI, USA). The total extract of the culture bottles was lysed via two or three cycles of freezing (–70 °C, 30 min) and thawing (37 °C, 12 min), followed by treatment with NP40 0.5% for 10 min. The lysates were collected in 250 mL culture bottles and centrifuged at 10,000 rpm (Rotor SLA-3000, ThermoFisher, Waltham, MA, USA) for 1 h at 20 °C. Next, the supernatants were collected and placed into culture bottles, where 50% of the total volume of a solution of PEG 20%/NaCl 0.2 M was added. The viral particles were precipitated for 1 h on ice, and were then centrifuged at 10,000 rpm (Rotor SLA-3000, ThermoFisher, Waltham, MA, USA) for 1 h at 20 °C. The supernatants were discarded and the precipitated virus was dissolved in 5 mL of CsCl (1.10 g/mL), and placed into tubes with CsCl at 1.3 and 1.4 g/mL densities, followed by ultracentrifugation at 20,000 rpm (Rotor Beckman Coulter Sw40Ti, Beckman Coulter, Brea, CA, USA) for 2 h at 20 °C. The adenovirus of interest was found between the 1.3 and 1.4 g/mL gradient interface and passed through a CL-4B Sepharose column (Sigma-Aldrich, St. Louis, MO, USA), with purified adenovirus finally recovered.

### 4.4. Adenoviral Titration via the Plaque-Forming Units Method

Human embryonic kidney 293A cells were cultured in petri dishes until 100% confluence was reached. The adenoviral particles were diluted with DMEM 1X supplemented with 2% FBS and penicillin/streptomycin (Gibco, Waltham, MA, USA). Subsequently, the 293A cells were infected with 1 mL of diluted viral particles for 1 h and placed into Petri dishes. The medium was removed and noble agar at 1.6% (Sigma-Aldrich, St. Louis, MO, USA) was added. The dishes were incubated at 37 °C with 5% CO_2_ and observed for seven days. To calculate the plaque-forming units (PFUs), the plaque number was multiplied by the dilution factor.

### 4.5. Electron Microscopy Analyses

An amount of 50 × 10^12^ adenoviral particles was placed into copper formvar-covered grids and fixed for 1 min. The grids were treated with negative staining with uranyl acetate 1X for 30 s. The samples were examined using a JEOL JEM-1010 transmission electron microscope (JEOL LTD, Tokyo, Japan) at 60 kV acceleration voltage. The images were obtained at 75,000× and 250,000× magnification.

### 4.6. Detection of Adenoviral Infection in Normal Skin

The mice were injected intradermally with adenoviral vector AdGFP at three different doses: 1 × 10^6^, 1 × 10^8^ and 1 × 10^10^ plaque forming units. After 24, 48 and 72 h post-injection, the relative light units (RLU) of GFP expression were measured using an IVIS Lumina III (Perkin Elmer, Waltham, MA, USA).

### 4.7. Imiquimod-Induced Psoriasis-like Skin Inflammation Model and Treatment with Adenoviral Constructions

Eleven groups of Balb/c mice with four animals per group were used as the imiquimod-induced psoriasis models. All mice were depilated on the back and treated as follows: group 1 received no treatment; groups 2–11 were treated topically with 30 mg of imiquimod 5% cream (Aldara, 3M Pharmaceuticals, Maplewood, MN, USA) for six days to induce psoriasiform lesions. Group 2 received no further treatment; group 3 received an intradermal (ID) injection with 100 uL of saline solution in four regions of the back (25 uL each one) 24 h prior to imiquimod application; groups 4 to 7 received an ID injection with AdVHL at four different concentrations (10^4^, 10^6^, 10^8^ and 10^10^ PFU/100 uL, respectively) of saline solution 24 h prior to imiquimod treatment; and groups 8 to 11 received an ID injection with AdGFP at the same concentrations 24 h prior to imiquimod application. On all six days of the experiment, the mice were examined and their backs were photographed. At day seven, all the mice were euthanized using a CO_2_ chamber. For subcutaneous vascularity, the skin was observed using a stereo microscope with Motic Cam 5.0 MP. The images obtained were at 7.5× magnification and were analyzed with Motic Images Plus 2.0 software (Miotic, Hong Kong).

### 4.8. PCR and RT-PCR

Total RNA extracts from the skin of control mice and mice treated with AdVHL of AdGFP vectors were obtained using TRIzol reagent (Invitrogen, MA, USA), according to the manufacturer’s instructions. cDNA synthesis was performed via reverse transcription using a High-Capacity cDNA Reverse Transcription Kit (Invitrogen, MA, USA). The PCRs were performed with MyTaq red polymerase (Meridian Bioscience, Cincinnati, OH, USA) following the provider’s instructions, and β-actin was used as the endogenous control gene. Table 1 shows the primers used to analyze the expression of mRNA of interest.

### 4.9. Immunohistochemistry

The skin of control and treated mice was fixed in formol (10%) and tissues were embedded in paraffin. Next, sections of 3 μm were obtained (Microtome Leica RM225, Leica, Munich, Germany) and placed in charged glass slides (SuperFrost Plus Yellow, Thermo Scientific, Waltham, MA, USA). The paraffin was removed through a train of solvents and antigen heat recovery was performed with incubation at 120 °C for 20 min with citrate buffer at pH 6.0. The permeabilization of tissue was carried out with a solution that contained 10 mg/mL of bovine serum albumin, sodium azide 0.02%, Triton 0.5% (Sigma-Aldrich, St. Louis, MO, USA) and horse serum 5% (Gibco, Waltham, MA, USA). The primary antibodies used were anti-pVHL (1:500, Sc-5575, Santa Cruz, TX, USA) and anti-HIF-1α (1:1000, NB100-479, Novus Biologicals, Centennial, CO, USA), and the peroxidase anti-rabbit IgG secondary antibody (Abcam, Cambridge, UK) was incubated for 1 h. Finally, the slides were counterstained with hematoxylin–eosin and images were obtained on a Leica microscope at 20× magnification. Analyses of the cell infiltration and acanthosis of the skin slides were obtained using Leica Q500/W analyzer software (Leica, Munich, Germany).

### 4.10. Immunofluorescence

Mouse skin from control and AdVHL-treated animals was processed with methylbutane for 5 min, then placed in liquid nitrogen, embedded in OCT compound (Sakura Finetek, Torrance, CA, USA) and cryopreserved at −70 °C. Next, sections of 8 μm were obtained and placed in charged glass slides (SuperFrost Plus Yellow, Thermo Scientific, Waltham, MA, USA), and the tissues were fixed in 4% paraformaldehyde and rehydrated with a series of solvents. Heat antigen retrieval was performed using a citrate buffer at pH 6.0 for 20 min at 90 °C, and the tissue slides were blocked with a solution of BSA/FBS 5% and sodium azide 0.02% in PBS for 2 h. Primary homemade rabbit polyclonal antibody anti-adenovirus (1:1000), mouse monoclonal antibody anti-pVHL (1:40, sc-55506, Santa Cruz, TX, USA), homemade rat monoclonal antibody anti-IL-17 (1:40, TC11-18H10, BD Bioscience, Franklin Lakes, NJ, USA), mouse monoclonal antibody anti- HIF-1α (1:15, 241812, Minneapolis, MN, USA), and homemade rabbit polyclonal antibody anti-LDH (1:80, Abcam, Cambridge, UK) were incubated overnight, and the goat anti-rabbit IgG-PE secondary antibody (1:300, sc3739, Santa Cruz, TX, USA), Donkey Anti-Rabbit IgG-AF647 (1:150), Anti-Rat IgG-AF594, Anti-Mouse IgG-AF647 and Anti-Mouse IgG-AF488 secondary antibodies (all 1:150, Jackson ImmunoResearch, West Grove, PA, USA) were then incubated for 2 h. The nuclei were stained with Hoescht (1:2000, ThermoFisher, Waltham, MA, USA) for 10 min, and the slides were mounted with VectaShield (Vector Laboratories, Burlingame, CA, USA). Images were obtained on a Carl Zeiss LSM 5.0 PASCAL (Carl Zeiss, Munich, Germany) confocal microscope using a 20× objective lens. Additionally, some images were obtained on a Nikon Ti Eclipse inverted confocal microscope (Nikon Corporation, Minato, Tokio, Japan) using 20× objective lens, and magnification was performed either at 3.4× or with digital zoom. All confocal microscopy images were analyzed using FIJI imageJ software.

### 4.11. Western Blotting

Total extracts from the skin of control and AdVHL-treated mice were collected in RIPA lysis buffer (150 mM NaCl, 1% NP-40, 0.5% deoxycholic acid, 0.1% SDS and 50 mM Tris (pH 8.0)) supplemented with protease and phosphatase inhibitors (Sigma-Aldrich, St. Louis, MO, USA). The tissue was sonicated and spun to obtain proteins. Forty micrograms of protein was loaded and separated in PAGE-SDS gel and transferred to a nitrocellulose membrane. The immunoblot was performed using the primary antibodies anti-pVHL (1:1000, Sc-5575, Santa Cruz, TX, USA), anti-HIF-1α (1:1000, NB100479, Novus Biologicals, Centennial, CO, USA), anti-GADPH (1:1000, sc-47724, Santa Cruz, TX, USA) and anti-β-actin (1:1000, homemade monoclonal antibody provided by Centro de Investigación y Estudios Avanzados, Instituto Politécnico Nacional). Next, mouse anti-rabbit IgG-HRP secondary antibody (1:20,000, sc-2357, Santa Cruz, TX, USA) was incubated and revealed with a luminata system (Merck Millipore, St. Louis, MA, USA). The signals were detected using a ChemiDoc MP System (Bio-Rad, Hercules, CA, USA).

### 4.12. Flow Cytometry

Skin from the ears of the mice was treated with imiquimod as described above. Skin cell suspensions were obtained by enzymatic digestion with 0.25 mg/mL Liberase TL and 0.125 mg/mL DNAse (Sigma-Aldrich, St. Louis, MO, USA) for 45 min at 37 °C. They were then chopped and incubated under the same conditions with constant shaking. Enzymatic digestion was stopped with 0.5 μM EDTA, and the cell suspensions were filtered through a 70 μm strainer (Corning Inc, Corning, NY, USA), followed by the addition of 0.125 mg/mL DNAse (Sigma-Aldrich, St. Louis, MO, USA). Finally, the cells were washed, counted and stained as needed. To allow for counting, the cells were stained with anti-CD45-PECy7 (1:400, T3/2.3, BioLegend, San Diego, CA, USA) and DAPI (1:5000,ThermoFisher, Waltham, MA, USA), and immediately mixed with CountBright absolute counting beads (ThermoFisher, Waltham, MA USA), acquired for flow cytometry. Cell surface staining was performed using the following antibodies: anti-CD45-PECy7 (1:400, T3/2.3, BioLegend, San Diego, CA, USA), anti-Ly6G-Alexa Fluor 488 (1:200, 1A8, BioLegend, San Diego, CA, USA) and anti-CD4-APC (1:200, RM4-5, eBioscience, Waltham, MA, USA). LIVE/DEAD Fixable Aqua (1:400, ThermoFisher, Waltham, MA, USA) staining was included. To achieve intracellular staining, cell surface staining was first performed, followed by fixation and permeabilization using an intracellular fixation and permeabilization buffer set (ThermoFisher, Waltham, MA, USA) according to the manufacturer’s instructions. To stain the cytokines, the True-Nuclear transcription factor buffer set (BioLegend, San Diego, CA, USA) was used, according to the manufacturer’s instructions. Intracellular staining included anti-IL-17-PE (1:100, TC11-18H10, BD Bioscience, Franklin Lakes, NJ, USA). The cells were acquired in a BD FACSCanto II or BD FACSAria IIu system (Becton, Dickinson and company, Franklin Lakes, NJ, USA), and flow cytometry data was analyzed with FlowJo 8.7 software (Tree Star, Inc., Ashland, OR, USA).

### 4.13. Statistics

The normality of the results was determined using the Shapiro–Wilks test. The data are presented as the mean ± SEM (standard error of the mean). The results with two variables were evaluated with a one-way ANOVA test, followed by a multiparametric Tukey test for data with multiple variables. Non-parametric data are presented as median ± CI (confidence interval) and analyzed using the Mann–Whitney U test for two variables or the Kruskal–Wallis test for three or more variables. All statistical analyses were performed using Prism V5.0 software (GraphPad, San Diego, CA, USA), and statistical significance was considered to be * *p* < 0.05, ** *p* < 0.01 or *** *p* < 0.001.

## 5. Conclusions

In conclusion, our results show a reduction in pVHL and an enhancement in HIF-1α expression in psoriatic skin models, which strengthens previous findings in psoriasis patients. Importantly, the recovery of pVHL expression was helpful in controlling the skin psoriasis features induced by IMQ treatment, such as scale, erythema and epidermal acanthosis, as well as considerably diminishing angiogenesis and severity. This amelioration of the lesions induced by pVHL could be explained by negative regulation of the important molecules in psoriasis, such as HIF-1α, VEGF, LDH and TNF-α. Similarly, pVHL also affected T cells and, importantly, neutrophil recruitment to lesional skin, along with the production of IL-17 by this immune cell population. Our work revealed that adenoviral vectors could be a successful approach to inducing pVHL expression in psoriatic skin, and may contribute to the development of new treatment strategies. Together, our data strongly suggest that the von Hippel–Lindau protein has a beneficial role in the pathophysiology of psoriasis.

## Figures and Tables

**Table 1:** Primers used to perform RT-PCR technique.

Gene	Sequence: 5′ to 3′
VHL	Fwd: -CCAATAATGCCCCGGAAGG-
	Rev: -TCAAGGCTCCTCTTCCAGGTG-
IL-17A	Fwd: -CAGGGAGAGCTTCATCTGTGT-
	Rev: -GCTGAGCTTTGAGGGATGAT-
VEGF	Fwd: -CTTGCAGATGTGACAAGCCAA-
	Rev: -AGCAGCAGATATAAGAAAATGGCG-
TNF-a	Fwd: -TCTCATCAGTTCTATGGCCCAG-
	Rev: -GGGAGTAGACAAGGTACAACCC-
GADPH	Fwd: -CTACCCCCAATGTGTCCGTC-
	Rev: -GCCGTATTCATTGTCATACCAGG-
b-ACTIN	Fwd: -ATGTGGATCAGCAAGCAGGA-
	Rev: -AAAGGGTGTAAAACGCAGCTC-

## Data Availability

The datasets analyzed during the current study are available from the corresponding author on reasonable request.

## Supplementary Materials

The following supporting information can be downloaded at: https://www.mdpi.com/article/10.3390/ijms23095226/s1.
