# Supplementary material for: The Protective Role of pVHL in Imiquimod-Induced Psoriasis-like Skin Inflammation
Source: Int J Mol Sci. 2022 May 7;23(9):5226. doi: 10.3390/ijms23095226 (PMC9104378; doi:10.3390/ijms23095226)
Supplement: Supplementary file 1 [file ijms-23-05226-s001.zip › ijms-1637854-supplementary.pdf]

Supplementary figures

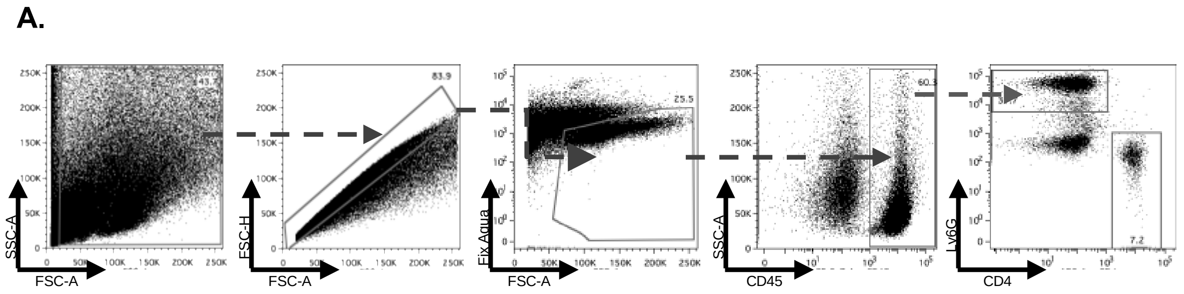

Supplementary Figure 1. Identification of T CD4 cells and neutrophils by flow cytometry. Psoriatic-like lesions were generated by imiquimod (IMQ) application in the ear of mice and received $1x10^{10}$ PFU of AdVHL vector. Skin cell suspensions were obtained as previously described. **(A)** Gating strategy excluding aggregates, dead cells (FixAqua) and identifying $CD45^{+}$ cells, neutrophils ($Ly6G^{+}$) and CD4+ T cells ($CD4^{+}$).
